# A Web-Based Intervention (MotivATE) to Increase Attendance at an Eating Disorder Service Assessment Appointment: Zelen Randomized Controlled Trial

**DOI:** 10.2196/11874

**Published:** 2019-02-27

**Authors:** James Denison-Day, Sarah Muir, Ciarán Newell, Katherine M Appleton

**Affiliations:** 1 Research Centre for Behaviour Change Psychology Department Bournemouth University Poole United Kingdom; 2 Dorset Healthcare University NHS Foundation Trust Poole United Kingdom

**Keywords:** feeding disorders, eating disorders, anorexia nervosa, bulimia nervosa, binge-eating disorder, motivation, early medical intervention, Internet

## Abstract

**Background:**

Early assessment and treatment of eating disorder patients is important for patient outcomes. However, up to a third of people referred for treatment do not access services and 16.4% do not attend their first scheduled assessment appointment. MotivATE is a fully automated, novel, Web-based program intended to increase motivation to change eating disorder behaviors, designed for delivery at the point of invitation to an eating disorder service, with the aim of increasing service attendance.

**Objective:**

This paper assesses the impact of MotivATE on attendance at assessment when compared with treatment-as-usual.

**Methods:**

A Zelen randomized controlled design was used. All individuals referred to a specialist eating disorder service, Kimmeridge Court in Dorset, UK, over the course of a year (October 24, 2016-October 23, 2017) were randomized to treatment-as-usual only or treatment-as-usual plus an additional letter offering access to MotivATE. Attendance at the initial scheduled assessment appointment was documented. Logistic regression analysis assessed the impact of MotivATE on attendance at assessment. Additional analyses based on levels of engagement with MotivATE were also undertaken.

**Results:**

A total of 313 participants took part: 156 (49.8%) were randomized to treatment-as-usual and 157 (50.2%) were randomized to receive the additional offer to access MotivATE. Intention-to-treat analysis between conditions showed no impact of MotivATE on attendance at assessment (odds ratio [OR] 1.35, 95% CI 0.69-2.66, *P*=.38). Examination of the usage data indicated that only 53 of 157 participants (33.8%) in the MotivATE condition registered with the Web-based intervention. An analysis comparing those that registered with the intervention with those that did not found greater attendance at assessment in those that had registered (OR 9.46, 95% CI 1.22-73.38, *P*=.03).

**Conclusions:**

Our primary analyses suggest no impact of MotivATE on attendance at the first scheduled assessment appointment, but secondary analyses revealed limited engagement with the program and improved attendance in those who did engage. It is unclear, however, if engagement with the program increased motivation and, in turn, attendance or if more motivated individuals were more likely to access the intervention. Further research is required to facilitate engagement with Web-based interventions and to understand the full value of MotivATE for users.

**Trial Registration:**

ClinicalTrials.gov NCT02777944; https://clinicaltrials.gov/ct2/show/NCT02777944 (Archived by WebCite at http://www.webcitation.org/75VDEFZZ4)

## Introduction

Eating disorders, including anorexia nervosa, bulimia nervosa, binge eating disorder, and other nonspecified feeding or eating disorders, are estimated to affect 725,000 people in the United Kingdom [1]. Eating disorders have the highest mortality rate of all mental health conditions [2] and can have devastating consequences for the affected individual and their family members [3], as well as for the wider community, with estimated costs to the National Health Service (NHS) of between £3.9 billion and £4.6 billion a year [1].

When treating eating disorders, early intervention can be vital, as this reduces the risks of chronicity, which in turn lowers the risks to the individual, the burden of care for families, and the costs to the health care system [4,5]. However, research highlights that up to a third of people referred for specialist psychological treatment do not access services [6].

Nonattendance is frequently due to a complex collection of factors, with individuals with eating disorders often being highly ambivalent about change [7-9]. The role of an individual's eating disorder can be highly valued and perceived as functional, for example, by providing a sense of emotional or social avoidance; in the early stages of an eating disorder, a person may be in denial about the problematic aspects of their behavior [10]. People can also become stuck in their behaviors, leading to low confidence and fears about change, as well as a perception of reduced control over their life and choices, resulting in a passive approach to treatment and recovery [11]. It has also been noted that people with eating disorders can hold negative preconceived expectations of what treatment will entail [11]. These internal factors are also compounded by external and practical issues, such as negative experiences with health care services and professionals, as well as the impact of social stigma. Qualitative research conducted with individuals recovering from eating disorders suggests that these factors among people with eating disorders are potentially modifiable barriers to engagement with services [9].

A service provision survey found that nonattendance at an eating disorders service, as a result of not opting into the service or not turning up to appointments, ranged from 10%-32% of referrals suitable for assessment [11]. As such, a novel Web-based intervention, MotivATE, was developed, which aimed to address some of these barriers and promote treatment engagement [11]. The intervention is intended for use between referral to an eating disorder service and assessment appointment; the intervention focuses on managing expectations of assessment, addressing ambivalence, and increasing users’ motivation and confidence to attend their initial appointment. This is achieved through the use of information, motivational tools, interactive activities, and stories from other individuals with eating disorders spread across four brief modules. MotivATE was developed via an iterative process of user evaluation through involvement of people that had experience with the assessment process. This was done within the framework of the intervention mapping process outlined by Bartholomew, Parcel, and Kok [12], which recognizes three phases of intervention development: needs assessment, program development, and evaluation. Full details of the intervention and development process are given elsewhere [11].

Following development, this single-center pilot study was conducted to establish an initial evidence base for the value of MotivATE in a naturalistic clinical setting. The aim of this research was therefore to test the impact of MotivATE on attendance at the first scheduled assessment appointment. The *principal research question* is as follows: Does adding MotivATE to treatment-as-usual impact on attendance at adult eating disorders outpatient services?

## Methods

### Design

The research was conducted using a two-arm, single-consent Zelen randomized controlled trial. A Zelen randomized consent design [13], which involves randomizing participants prior to consent and then only collecting consent from those in the active condition, was proposed as the most ethical and appropriate approach for answering the research question for several reasons. Firstly, people with eating disorders are often highly ambivalent about recovery [9]. As such, adding trial consent, particularly with the knowledge that they may not receive the active condition, at a time that might be challenging for this group could be deemed unethical. The addition of full consent prior to randomization ran the risk of resentful demoralization within the control group, potentially increasing the rate of nonattendance at assessment appointments. Hawthorne effects, which result in participants changing their behavior as a result of knowing they are being observed, may also occur [14]. Resentful demoralization and Hawthorne effects would not only have implications for the validity of the study, but may also pose significant risks to the health and well-being of the patient and their loved ones. As such, the use of a Zelen design reduces biases and potential negative outcomes. It also allows for a trial that more closely replicates anticipated procedures in usual clinical practice.

### Recruitment

No active recruitment took place for the study. All adult referrals to the Kimmeridge Court Eating Disorders service in Dorset, UK, over the period of one year (October 24, 2016-October 23, 2017) were identified for potential inclusion in the study. This time period was chosen to ensure that results were not affected by seasonal variations. No power calculations were done due to both the limited previous literature directly related to this research and the large degree of variation identified in figures for previous attendance rates across eating disorder services [11]. However, a post hoc power analysis was conducted and is presented in the discussion section. This analysis is intended to inform future similar studies. Participants were referred to the eating disorder service by a health professional as part of the usual referral process. Upon referral to the service, patient information was checked by the eating disorders service staff against the inclusion and exclusion criteria listed in Textbox 1.

All eligible referrals were then randomized into the study using the randomization procedure outlined below.

### Control Condition: Treatment-as-Usual

Participants randomized to the control condition received usual care. This consisted of a phone call from service center staff, with details of participants' assessment appointments; a letter; and a compliments, comments, concerns, and complaints leaflet sent to their home address.

### Intervention Condition: MotivATE

Participants randomized to the intervention condition received usual care, plus the opportunity to access MotivATE [11]. MotivATE is a fully automated Web-based intervention delivered via four 15-20-minute Web-based modules designed to be used prior to an assessment appointment. Participants were able to access MotivATE from their home computer or via tablet or mobile phone. The content and aims of these modules are briefly outlined in Table 1. Screenshots of different sections of MotivATE can be found in Multimedia Appendix 1.

Access to MotivATE was offered in addition to treatment-as-usual via an invitation letter to access MotivATE. The MotivATE invitation letter included a brief outline of MotivATE, the participant’s ID number to be used at registration, and the URL to access the intervention online.

Should a participant not access any modules within seven days of registering, they would receive an automated reminder email, with a second reminder being sent 14 days after registration. Similarly, after completing a module, should a participant not move on to another module, they would receive email reminders after seven and 14 days. Upon completion of all modules, participants received a final email congratulating them on completing MotivATE. The intervention was not altered in any way throughout the course of the trial.

### Outcomes

Our primary outcome was attendance at the initial assessment appointment. This was assessed using NHS audit data from the eating disorders service, which provided the number of individuals who did not attend from the MotivATE group versus the control group.

Secondary outcomes were as follows:

1. Engagement with the intervention. Engagement was examined using data on the number of sessions completed by each participant and time spent accessing them; this was generated by the intervention.
2. Participants’ perceptions of MotivATE and the perceived impact of MotivATE on their motivation to attend assessment. This was assessed using qualitative data collected from participants from the MotivATE group in semistructured interviews. Participants in this group were given the opportunity to opt in to take part in a semistructured qualitative interview upon registering with the intervention. This triggered an automated email following the participants’ assessment appointment that outlined the details of the interview and invited participants to take part. At the midpoint of the study, a second follow-up email, sent out two weeks after the initial invitation, was also added in an attempt to improve uptake.

Due to the nature of the study design, no baseline measures or demographic data were collected.

### Initial Assessment Appointment

The initial assessment for both conditions was the same, constituting usual care, with attendance at this appointment being assessed using routine audit data; data regarding assessment attendance was added to the secure participant database by service staff. All treatment following the initial assessment was usual care and beyond the scope of this study.

### Randomization, Allocation Concealment, and Blinding

Study-relevant information (ie, control or intervention) was placed in opaque envelopes labeled with participant ID numbers. Participants were prerandomized using block randomization into the intervention or control arms by a member of the research team not directly involved in conducting the study (KMA). This was achieved by generating a random number string from Random.org [15], which was then broken down into consecutive blocks of eight digits and manually balanced to ensure even allocation in each block.

As participants were referred to the service, they were assigned a participant ID incrementally by the service center staff. The opaque envelopes labeled with participant numbers were then included with the invitation-to-assessment letter by service staff. All service center staff and researchers were thus blind to each referral’s group allocation, while participants were blind to other possible conditions.

**Inclusion and exclusion criteria.**

Inclusion criteria:

1. Referrals to the eating disorders service during the study period.

Exclusion criteria:

1. Inpatients or cases that are deemed to be an emergency (ie, must be seen within one day) or urgent (ie, must be seen within seven days).
2. Patients who have already been randomized into the study.
3. Non-English speakers.

**Table 1:** Content of the MotivATE Web-based intervention.

Module	Aim	Description
1: What happens at the first appointment?	Address expectations about the assessment appointment	Provides an interactive quiz to explore common misconceptions about assessment, information about the assessment appointment, and stories and videos about others’ experiences.
2: How motivated are you?	Introduce the idea of change	Introduces people to the stages-of-change model with stories of others’ experiences. User can choose their stage of change.
3: Arming yourself with information	Help with recognizing problematic behaviors (precontemplation) Address ambivalence	Information about eating disorders that relate to the pros and cons of eating disorders. Those who have selected a contemplation or preparation stage of change can complete their own pros and cons table and complete exercises designed to address ambivalence. Again, stories of others’ experiences of an eating disorder are included.
4: Preparing for your assessment	Improve confidence to attend	Includes a video of a clinician welcoming them to the assessment and allows users to make plans to attend their appointment.

Service staff maintained a single, secure record linking the participant’s name to their unique participant ID. Once all participants had passed through the study, outcome data was added to the secure record by service staff and any personal identifiers removed prior to this document being delivered to the research team.

### Analysis

As outcome data were collected for all participants, due to the nature of the study design, a complete analysis was conducted on an intention-to-treat basis with all data categorized according to the original allocation, using a two-sided 5% significance level. To examine the research question, a logistic regression was conducted.  The independent variable for this analysis was the allocated condition (*MotivATE* or *treatment-as-usual*); the dependent variable was attendance at assessment (*attended* or *did not attend*). 

Usage data from the MotivATE condition was analyzed using descriptive statistics in order to explore the number of sessions registered by MotivATE, which includes any visit to the intervention, participants who registered with the intervention, as well as how many of the intervention modules were completed by users. Additionally, a follow-up of our primary analysis was conducted to determine the attendance of participants within the MotivATE condition who registered, or did not register, with MotivATE.

Qualitative data were originally to be analyzed using thematic analysis [16]. However, as a result of poor recruitment to this aspect of the study, it was not possible to complete a full analysis of the data. Data were analyzed as case studies instead.

### Ethical Approval

The research gained Health Research Authority approval (Reference: 16/SC/0431) from the Hampshire A Ethics Committee and was registered at ClinicalTrials.gov (NCT02777944) prior to commencement. In order to ensure participant safety, all researchers completed Good Clinical Practice (GCP) training and adhered to standard NHS practice guidelines.

## Results

### Primary Outcome: Attendance at Initial Assessment Appointment

**Figure 1:**
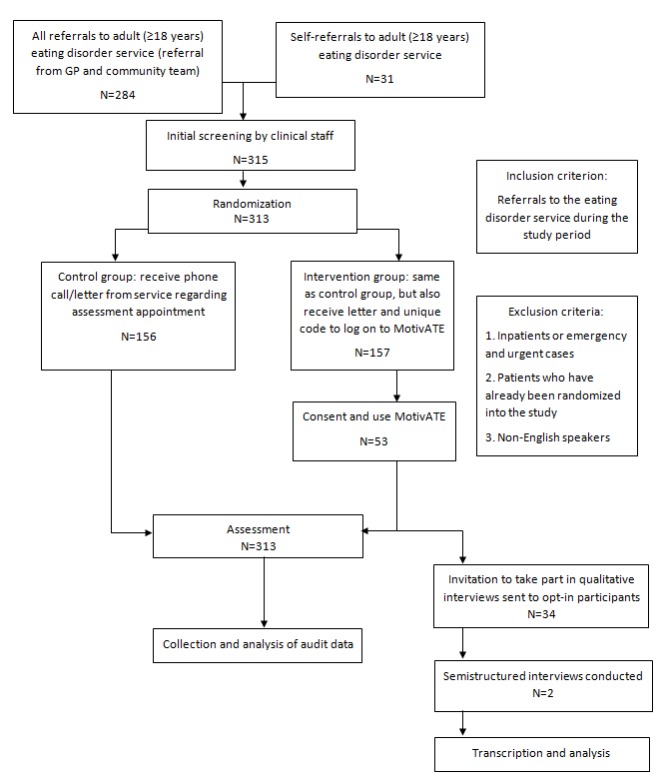
CONSORT flow diagram. GP: general practitioner.

Figure 1 shows the flowchart of the trial. In total, 315 participants were identified for recruitment; however, as a result of a replication in the randomization process, 2 participants were not randomized. This resulted in 313 participants randomized into the trial, of which 157 (50.2%) were randomized to the MotivATE condition and 156 (49.8%) were assigned to the control condition. In total, 274 out of 313 (87.5%) participants attended their assessment appointment, as shown in Table 2.

Binary logistic regression indicated no differences between groups in attendance at the assessment appointment (χ^2^_1_=0.8, *P*=.38). The odds ratio (OR) for the effect of being offered access to MotivATE on attendance was 1.35 (95% CI 0.69-2.66).

**Table 2:** Distribution of attendance at assessment appointment across study groups.

Condition	Attended assessment, n (%)	
	Yes	No
MotivATE (N=157)	140 (89.2)	17 (10.8)
Control (N=156)	134 (85.9)	22 (14.1)

**Table 3:** Number of participants that completed each module.

Module	Number of users that completed the module (N=53), n (%)
1	45 (85)
2	36 (68)
3	27 (51)
4	24 (45)

**Table 4:** Distribution of attendance at assessment for those that did and did not access MotivATE.

Condition	Attended assessment, n (%)	
	Yes	No
Accessed MotivATE (N=53)	52 (98)	1 (2)
Did not access MotivATE (N=104)	88 (84.6)	16 (15.4)

### Secondary Outcome: Engagement With the Intervention

Usage analysis shows that, of the 157 participants assigned to the MotivATE condition, only 53 (33.8%) registered with the intervention. During the trial period, MotivATE registered 1280 separate sessions; however, of these, only 87 (6.80%) were sessions generated by the 53 registered participants. As it was not possible to collect data for nonregistered users, the exact nature of the additional 1193 sessions is difficult to ascertain. However, while some of these are likely to have been participants that visited the intervention but did not register, the high number in relation to recruitment would suggest that this also includes visits by web crawlers, programs, or automated scripts that browse the Internet in a methodical, automated manner. Indeed, 1177 of these sessions consisted of visits to the homepage of one second or less. Of the 53 participants that registered with MotivATE, 8 (15%) completed registration but did not engage with any of the content. The remaining 45 participants (85%) went on to complete, on average, at least half of the available content, with the average participant completing two and a half of the MotivATE modules; 24 of the 53 registered participants (45%) completed all of the four modules. Table 3 shows a breakdown of the number of participants that completed each module. Of the 53 participants that accessed MotivATE, 17 (32%) used the intervention more than once, with the average across all participants being 1.64 visits per person.

Table 4 gives the distribution of attendance at assessment for those that did and did not access MotivATE.

Binary logistic regression indicated that registration with MotivATE did act as a significant predictor of attendance at assessment, where individuals who registered with MotivATE were 9.5 times more likely to attend than those who did not (OR 9.46, 95% CI 1.22-73.38, *P*=.03). In this instance, the model was found to significantly predict attendance at assessment (χ^2^_1_=8.5, *P*=.004).

### Secondary Outcome: Perceptions of the Intervention

Only 2 individuals of the 53 (4%) users who registered with MotivATE volunteered for and undertook an interview regarding their perceptions of MotivATE. These interviews have been written up as case studies and are provided in Multimedia Appendix 2.

In brief, the two case studies presented conflicting views of the usefulness of MotivATE to the participants. In one case, MotivATE did appear to have fulfilled its intended purpose; it helped to alleviate anxieties, addressed concerns, and provided practical help to assist in attending the assessment appointment. However, the other case related a much more negative experience, suggesting that the intervention felt patronizing in its approach and reinforced negative preconceptions. Two aspects, however, were common across both participants. First, both interviewees raised concerns that MotivATE represented a “tick-box” exercise rather than a genuine and helpful tool; this was a concern that was alleviated for one participant upon using MotivATE, but was exacerbated for the other. Second was an opinion that, even if MotivATE was not specifically beneficial, the underlying idea was a positive one.

## Discussion

This initial research indicates that, at present, MotivATE does not increase attendance at an initial assessment appointment in people with eating disorders. Secondary analyses, however, do give some insight into the potential impact of the intervention. Attendance was 9.5 times more likely for those who registered with MotivATE than those who did not, but registration with the intervention was low (53/157, 33.8%). This may suggest that the lack of overall impact in the study was not a result of the lack of impact of the intervention itself, but rather due to issues of uptake.

Problems with engagement are often encountered when rolling out online interventions [17] and it has been noted that participants of Internet interventions can exhibit lower levels of engagement than program developers originally envision [18]. However, Web-based interventions have been demonstrated to successfully encourage behavior change for a variety of health behaviors and among a variety of populations [19-21]. Lack of engagement, though, may be a particular issue for people with eating disorders. Several recent studies of online motivational interventions for eating disorders similarly found issues with engagement and high dropout [22-25]. This issue has been attributed to the poor ability of online interventions to attract and retain visitors, relative to other modes of contact [26].

Digital interventions have been identified as a potential approach to improving motivation to change among people with eating disorders [27]; our findings do not rule out this approach to addressing issues regarding low levels of treatment attendance. Indeed, both participants in the case studies suggested that, while MotivATE may not in and of itself be the solution, at least in its current form, the underlying concept remained a positive one. Indeed, once registered, the majority of participants completed more than half of the MotivATE content and a third visited more than once. The key issue faced by MotivATE, which was designed to promote treatment uptake, was itself a lack of uptake. This suggests that approaches addressing low engagement may in fact need to be considered even earlier in the treatment pathway and that simply offering new interventions to address this problem may not be enough. Rather, the way in which these interventions, and indeed treatment more broadly, are presented may need to be more deeply considered.

Furthermore, the observed rate of attendance at initial assessment appointments across this study was unusually high, with nonattendance levels of only 14% recorded for the control group. While within the previously noted range of 10%-32% [11], this figure represented the lower end of this range. Indeed, examination of previous nonattendance rates for the service in the two years prior to the study indicated nonattendance levels of 21% (2014-2015) and 20% (2015-2016). This low level of nonattendance may have impacted the outcome of the primary research question, as this reduced the scope for potential improvements as a result of introducing MotivATE. While it is possible that this change in attendance in comparison to previous years is a result of natural fluctuations, this change may also suggest that other factors present during the study period may have impacted treatment attendance. For example, it is possible that knowledge among service staff that a new intervention was being offered to some patients may have positively influenced the overall service provided, such as the nature of interactions during the phone consultations. It is also possible that this may have been influenced by factors outside of the service, such as changes in local practices relating to the referrals of individuals with eating disorders or initiatives by local charities. However, without further research it is difficult to pinpoint an exact cause.

This study had a number of strengths that support the findings outlined above. The high level of involvement of patients and the public in the development of MotivATE in the early stages ensured that the intervention was designed using a particularly person-based approach. The naturalistic nature of the design means that the research reflects the conditions found in practice. Similarly, conducting the study over one year eliminated the impact of seasonal fluctuations. A further strength of this research was the inclusion of qualitative interviews, which allowed for a greater insight into participants' perspectives of the intervention and its potential impact. The low recruitment to the interviews also provides useful feedback that reliance on email contact to recruit individuals from this population to an interview study appears to be insufficient and more salient approaches should be favored in future research.

Despite these strengths, this study did suffer from a number of limitations. Firstly, the use of only one site limits the applicability of the findings to general practice. This is due to different sites having differing treatment approaches and procedures, such as opt-in programs or longer waiting times, which may impact the effectiveness of the intervention. A second limitation was the lack of a pre-post measure of motivation to change. This was not included due to the naturalistic design of the study; however, without this it is not possible to ascertain if the intervention did improve motivation to change as intended. Motivation to change may have improved, but not sufficiently so to translate into behavior. Alternatively, improved attendance among those who registered for MotivATE may demonstrate increased motivation to change as a result of using MotivATE; it may also suggest that those with a higher motivation to change were more likely to attend anyway and, in turn, more likely to engage with the intervention [28]. Additionally, this study also included individuals who self-referred and these people are likely to have been more motivated to attend than those referred by general practitioners and other health care professionals; however, these individuals constituted less than 10% of the sample and were randomized across both groups; therefore, they are unlikely to have impacted on differences between the conditions. A final limitation is a potential lack of power in the analysis of the primary research question. A post hoc power analysis conducted in G*Power (Heinrich-Heine-Universität Düsseldorf) indicated an achieved power of only .37 in our study. In order to successfully detect an effect at the level observed, at a power of .8 and an alpha of .05, a further power analysis suggests a required sample size of 2559 (OR 1.35, Pr[Y=1 | X=1] H0=0.86). The clinical impact of an effect of this size, however, would also need to be considered.

This work highlights a number of directions for future research. Specifically relating to MotivATE, it is clear that more research is needed to understand the impact that the intervention has on its users, something that should be pursued from both quantitative and qualitative perspectives. This would allow for the investigation of not just whether MotivATE improves outcome measures such as motivation to change, but also some of the personal perspectives and issues surrounding engagement. A better understanding of how to best engage people with eating disorders with interventions, both on- and offline, is needed as early as possible in the treatment pathway. At present, this is not adequately understood and, as such, future research to explore potential ways in which engagement and uptake might be improved would be beneficial. Further research into uptake and engagement with interventions in a general sense is also required. New interventions are developed on a regular basis, but changes to behavior do not always follow.

In conclusion, in its current state, MotivATE cannot be recommended as an intervention to address lack of attendance at eating disorder services. However, with further research and development, this does not rule out the use of digital interventions as a potential approach to addressing this issue.

## Appendix

Multimedia Appendix 1 MotivATE screenshots.

Multimedia Appendix 2 MotivATE case studies.

Multimedia Appendix 3 CONSORT‐EHEALTH checklist (V 1.6.1).

## Abbreviations

GCP: Good Clinical Practice
GP: general practitioner
NHS: National Health Service
OR: odds ratio

## References

[1] BEAT (Beating Eating Disorders) (2015). The Costs of Eating Disorders: Social, Health and Economic Impacts.

[2] Arcelus J, Mitchell AJ, Wales J, Nielsen S (2011). Mortality rates in patients with anorexia nervosa and other eating disorders. A meta-analysis of 36 studies. Arch Gen Psychiatry.

[3] Nielsen S, Bará-Carril N, Treasure J, Schmidt U, van Furth E (2003). Family, burden of care, and social consequences. Handbook of Eating Disorders. 2nd edition.

[4] Le Grange D, Loeb KL (2007). Early identification and treatment of eating disorders: Prodrome to syndrome. Early Interv Psychiatry.

[5] Vaz AR, Conceição E, Machado PP (2014). Early response as a predictor of success in guided self-help treatment for bulimic disorders. Eur Eat Disord Rev.

[6] Waller G, Schmidt U, Treasure J, Murray K, Aleyna J, Emanuelli F, Crockett J, Yeomans M (2018). Problems across care pathways in specialist adult eating disorder services. Psychiatr Bull.

[7] Williams S, Reid M (2010). Understanding the experience of ambivalence in anorexia nervosa: The maintainer's perspective. Psychol Health.

[8] Colton A, Pistrang N (2004). Adolescents' experiences of inpatient treatment for anorexia nervosa. Eur Eat Disord Rev.

[9] Leavey G, Vallianatou C, Johnson-Sabine E, Rae S, Gunputh V (2011). Psychosocial barriers to engagement with an eating disorder service: A qualitative analysis of failure to attend. Eat Disord.

[10] Schoen E, Lee S, Skow C, Greenberg ST, Bell AS, Wiese JE, Martens JK (2012). A retrospective look at the internal help-seeking process in young women with eating disorders. Eat Disord.

[11] Muir S, Newell C, Griffiths J, Walker K, Hooper H, Thomas S, Thomas PW, Arcelus J, Day J, Appleton KM (2017). MotivATE: A pretreatment Web-based program to improve attendance at UK outpatient services among adults with eating disorders. JMIR Res Protoc.

[12] Bartholomew LK, Parcel G, Kok G (1998). Intervention mapping: A process for developing theory- and evidence-based health education programs. Health Educ Behav.

[13] Zelen M (1979). A new design for randomized clinical trials. N Engl J Med.

[14] Payne G, Payne J (2004). Key Concepts in Social Research.

[15] Random.org.

[16] Braun V, Clarke V (2006). Using thematic analysis in psychology. Qual Res Psychol.

[17] Doherty G, Coyle D, Sharry J (2012). Engagement with online mental health interventions: An exploratory clinical study of a treatment for depression. Proceedings of the SIGCHI Conference on Human Factors in Computing Systems.

[18] Eysenbach G (2005). The law of attrition. J Med Internet Res.

[19] Heber E, Ebert DD, Lehr D, Cuijpers P, Berking M, Nobis S, Riper H (2017). The benefit of Web- and computer-based interventions for stress: A systematic review and meta-analysis. J Med Internet Res.

[20] Watson S, Woodside JV, Ware LJ, Hunter SJ, McGrath A, Cardwell CR, Appleton KM, Young IS, McKinley MC (2015). Effect of a Web-based behavior change program on weight loss and cardiovascular risk factors in overweight and obese adults at high risk of developing cardiovascular disease: Randomized controlled trial. J Med Internet Res.

[21] Wantland DJ, Portillo CJ, Holzemer WL, Slaughter R, McGhee EM (2004). The effectiveness of Web-based vs non-Web-based interventions: A meta-analysis of behavioral change outcomes. J Med Internet Res.

[22] Hötzel K, von Brachel R, Schmidt U, Rieger E, Kosfelder J, Hechler T, Schulte D, Vocks S (2014). An Internet-based program to enhance motivation to change in females with symptoms of an eating disorder: A randomized controlled trial. Psychol Med.

[23] Leung SF, Ma LC, Russell J (2013). An open trial of self-help behaviours of clients with eating disorders in an online programme. J Adv Nurs.

[24] Leung SF, Ma J, Russell J (2013). Enhancing motivation to change in eating disorders with an online self-help program. Int J Ment Health Nurs.

[25] Leung SF, Ma JL, Russell J (2013). Enhancing quality of life in people with disordered eating using an online self-help programme. J Eat Disord.

[26] Lin JCC (2007). Online stickiness: Its antecedents and effect on purchasing intention. Behav Inf Technol.

[27] Denison-Day J, Appleton KM, Newell C, Muir S (2018). Improving motivation to change amongst individuals with eating disorders: A systematic review. Int J Eat Disord.

[28] Prochaska JO, Gellman MD, Turner JR (2013). Transtheoretical model of behavior change. Encyclopedia of Behavioral Medicine.

