# Supplementary material for: A Web-Based Intervention (MotivATE) to Increase Attendance at an Eating Disorder Service Assessment Appointment: Zelen Randomized Controlled Trial
Source: J Med Internet Res. 2019 Feb 27;21(2):e11874. doi: 10.2196/11874 (PMC6414822; doi:10.2196/11874)

Appendix 1 MotivATE Screenshots

Front Page

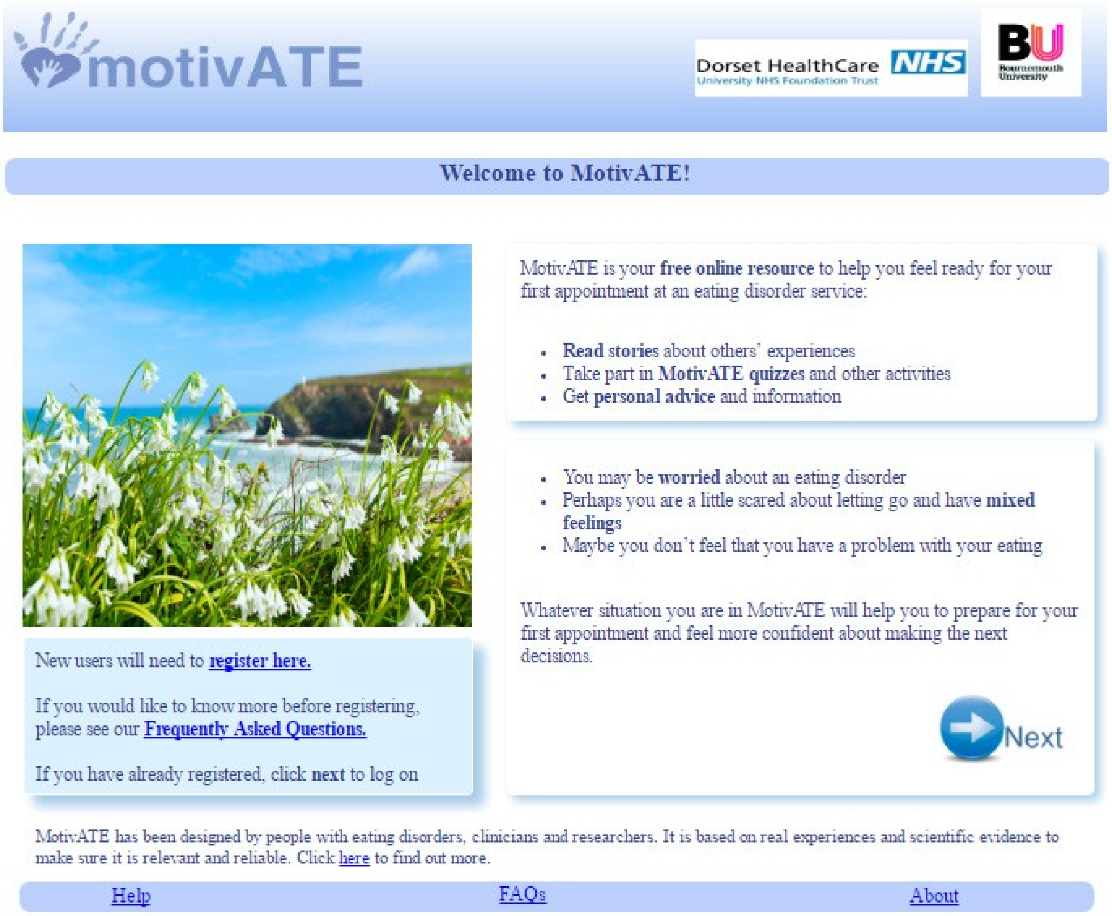

Module Home Page

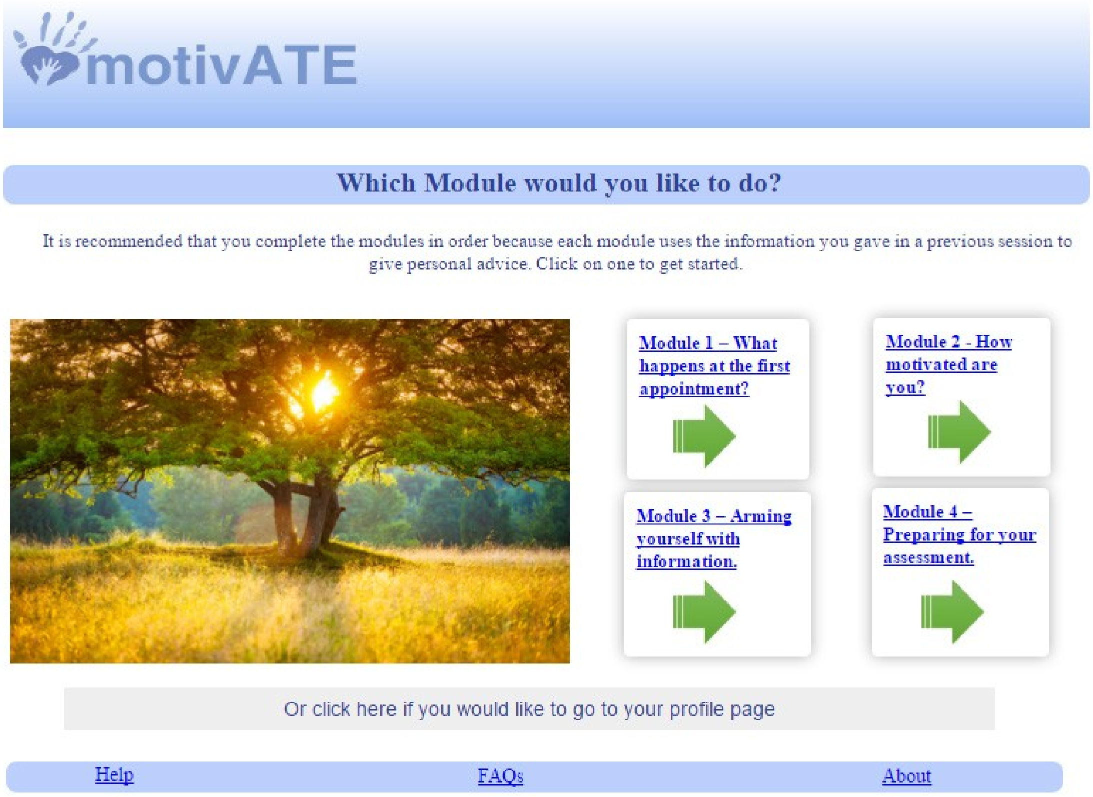

Module 1 Start

motivATE

Welcome to Module 1

This session answers the question: **"What happens at my assessment?"**

"Sometimes I hate my eating disorder and just want to recover. Other times I can't see a way to live my life without it. I'm worried about how I will feel on the day of my assessment." (Lilly, 21)

- Maybe this sounds familiar. **You may find it difficult thinking about life without an eating disorder.** Perhaps you don't even know how to change or don't think you need to change at all.
- **These feelings are completely natural but can mean that you may be in two minds about going to your assessment.** MotivATE can help you to feel ready to discuss these feelings with a clinician.

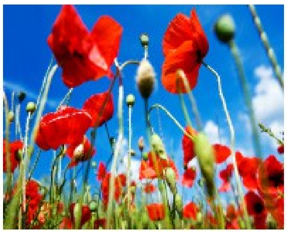

If at any point you feel any distress or drop in mood, please click on the "Help" button at the bottom of every page. Also, don't forget that you can leave a module at any time or take a break.

Back Next

Help FAQs About

Module 1 Survey

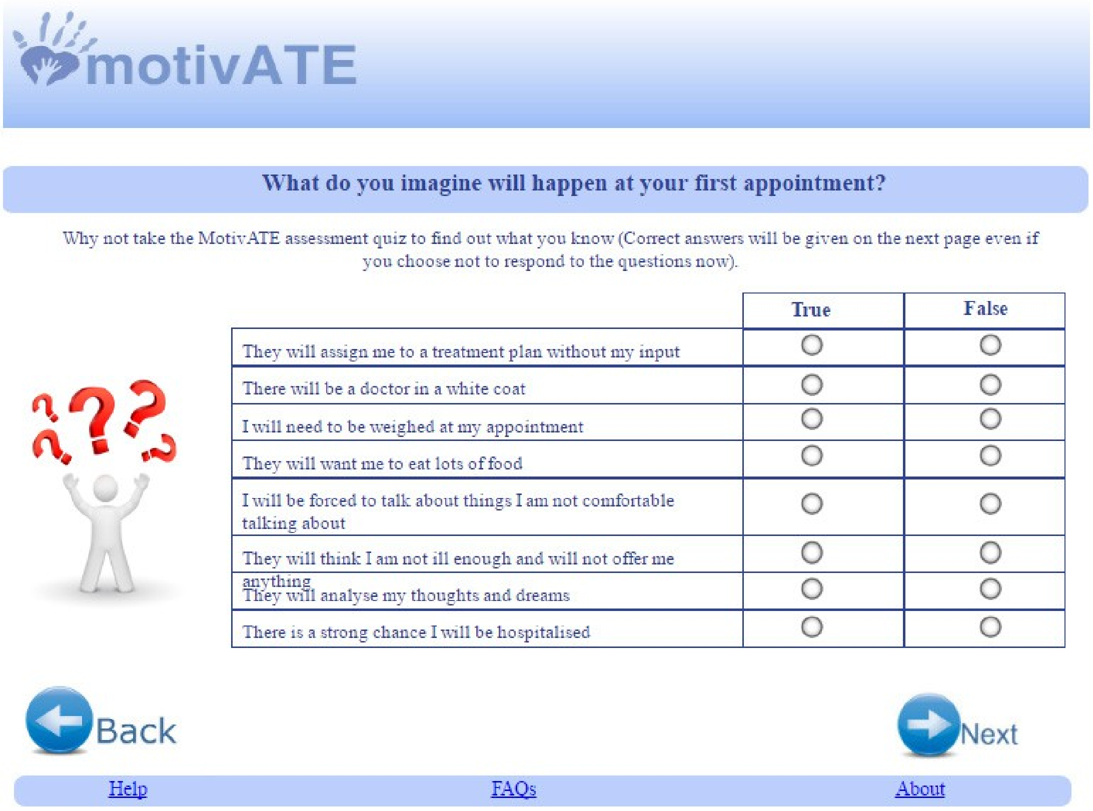

motivATE

What do you imagine will happen at your first appointment?

Why not take the MotivATE assessment quiz to find out what you know (Correct answers will be given on the next page even if you choose not to respond to the questions now).

	True	False
They will assign me to a treatment plan without my input	○	○
There will be a doctor in a white coat	○	○
I will need to be weighed at my appointment	○	○
They will want me to eat lots of food	○	○
I will be forced to talk about things I am not comfortable talking about	○	○
They will think I am not ill enough and will not offer me anything	○	○
They will analyse my thoughts and dreams	○	○
There is a strong chance I will be hospitalised	○	○

Back Next

Help FAQs About

Module 1 Survey Answers

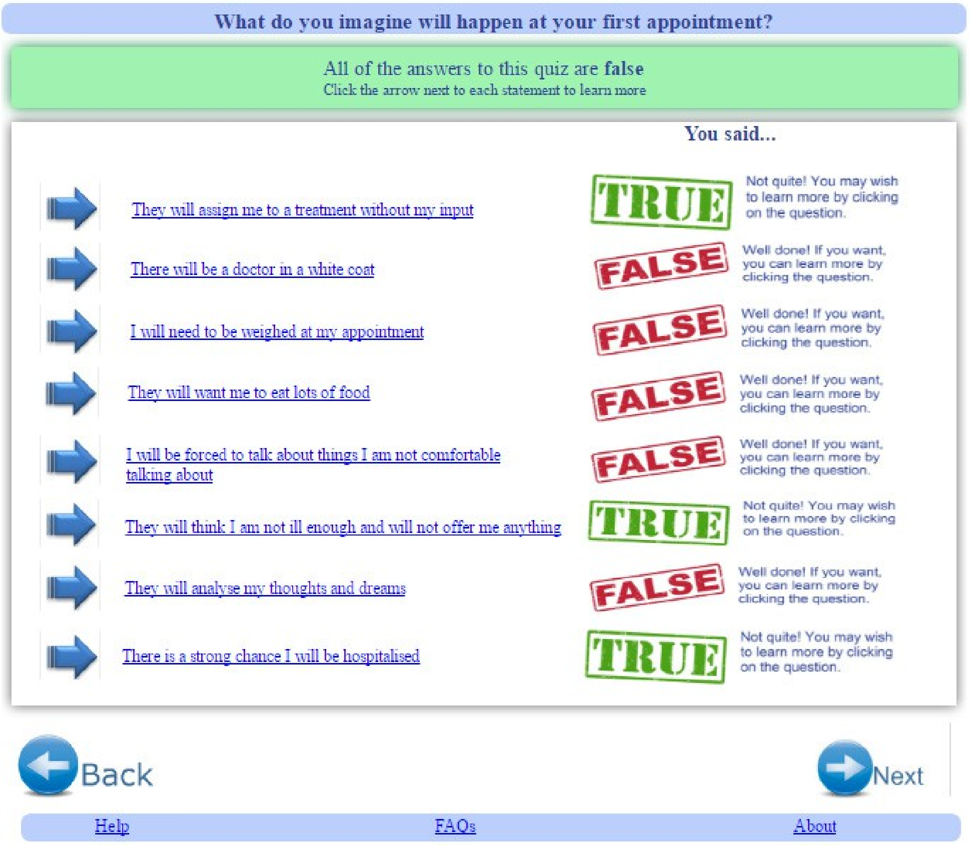

Module 1 Questions For Assessment

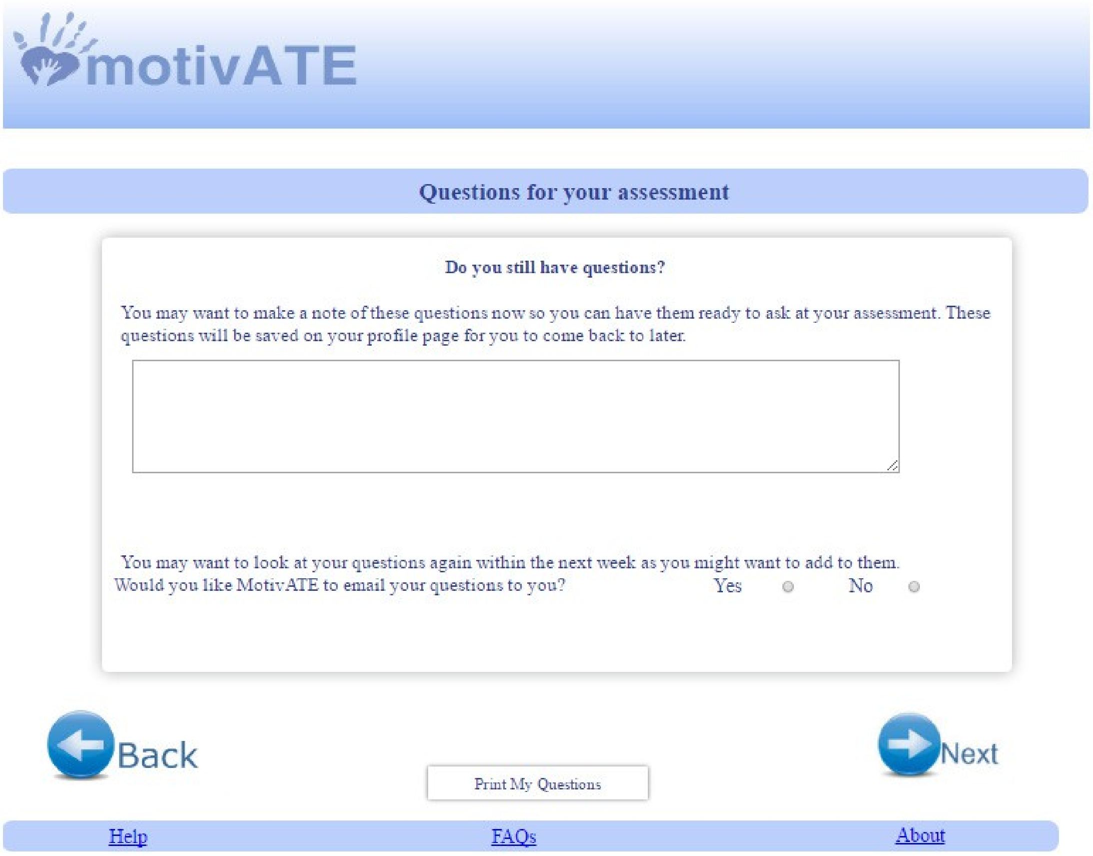

Module End (similar page is shown at end of all modules)

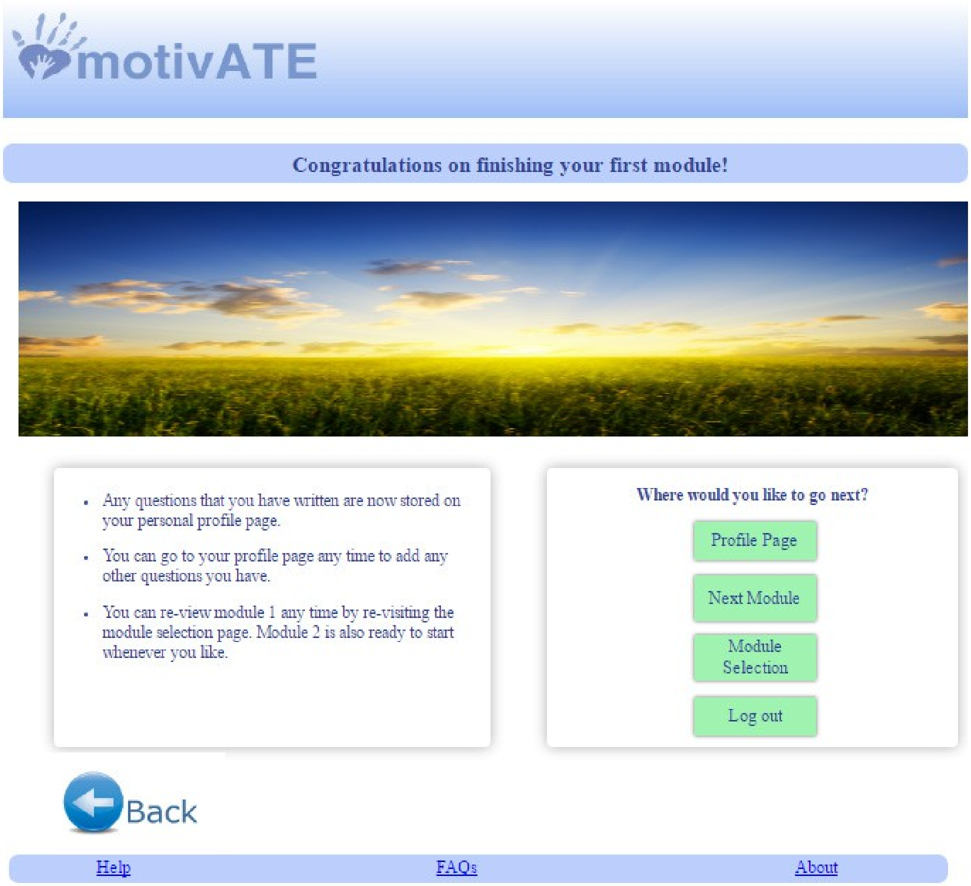

Module 2 Start

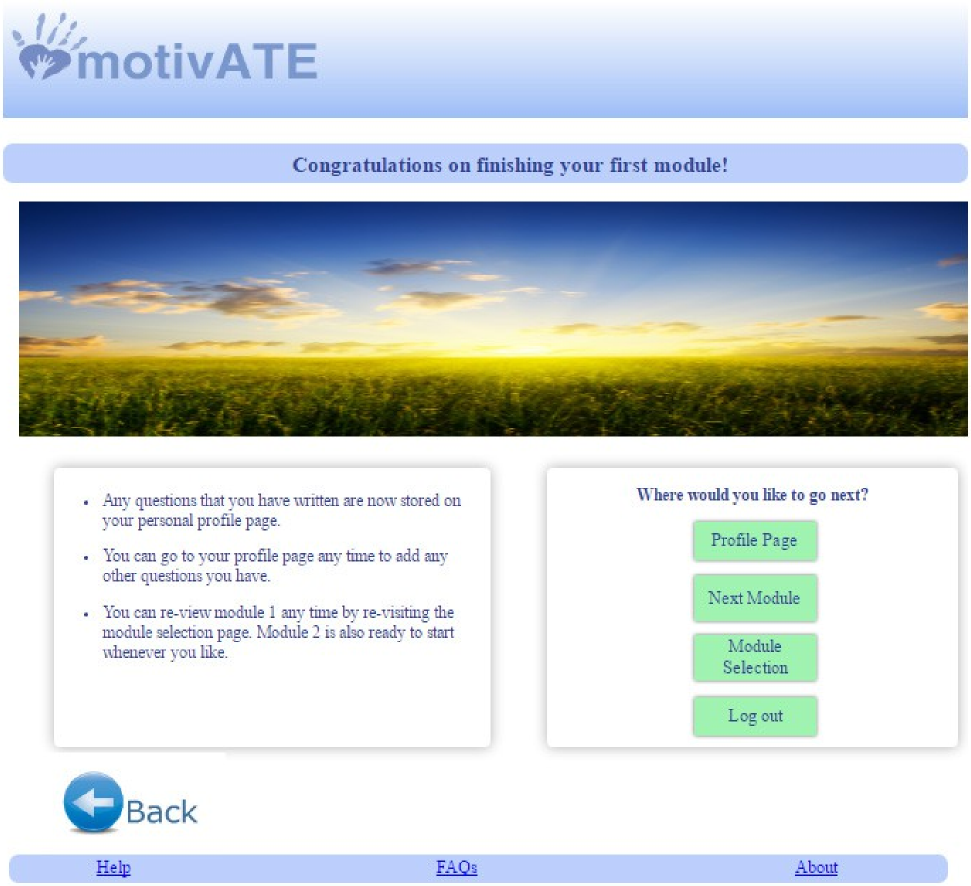

Module 2 Stage Choice 1

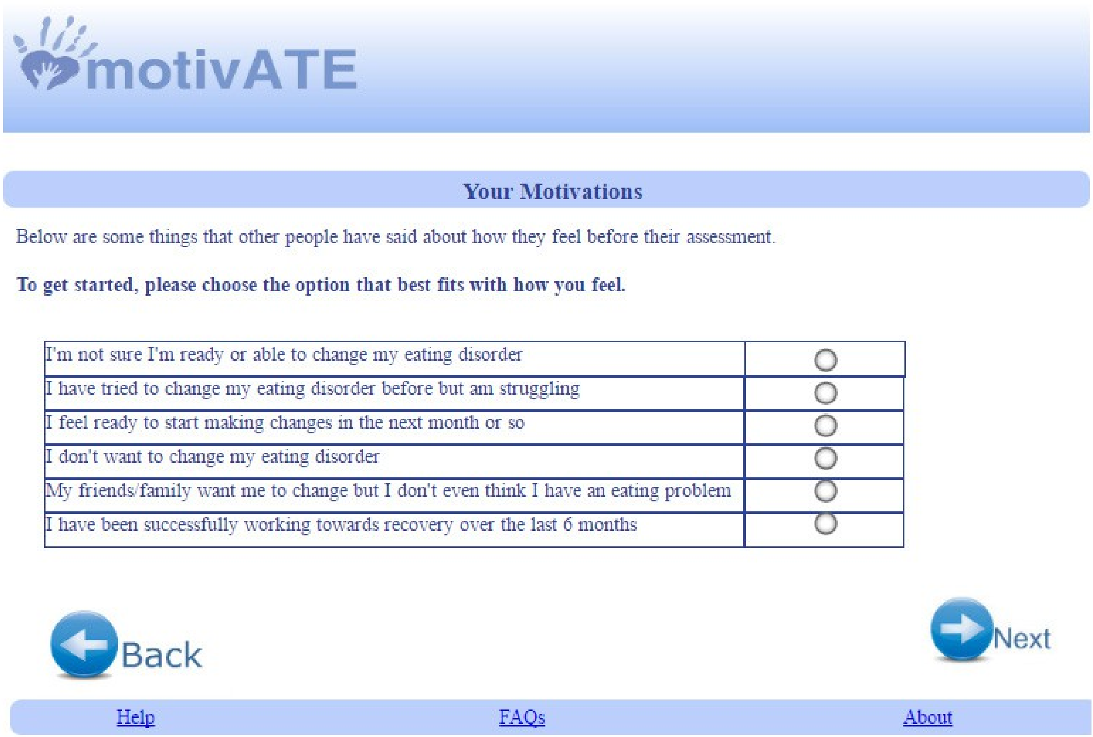
motivATE

Your Motivations

Below are some things that other people have said about how they feel before their assessment.

To get started, please choose the option that best fits with how you feel.

I'm not sure I'm ready or able to change my eating disorder	○
I have tried to change my eating disorder before but am struggling	○
I feel ready to start making changes in the next month or so	○
I don't want to change my eating disorder	○
My friends/family want me to change but I don't even think I have an eating problem	○
I have been successfully working towards recovery over the last 6 months	○

Back

Next

Help FAQs About

Module 2 Stages Information

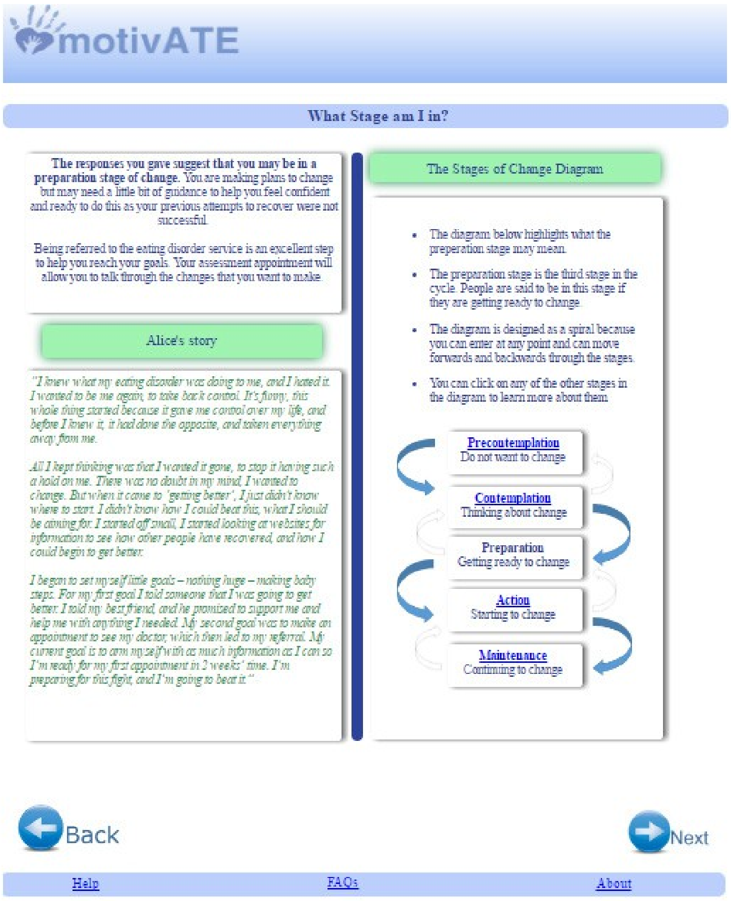

Module 2 Stage Choice 2

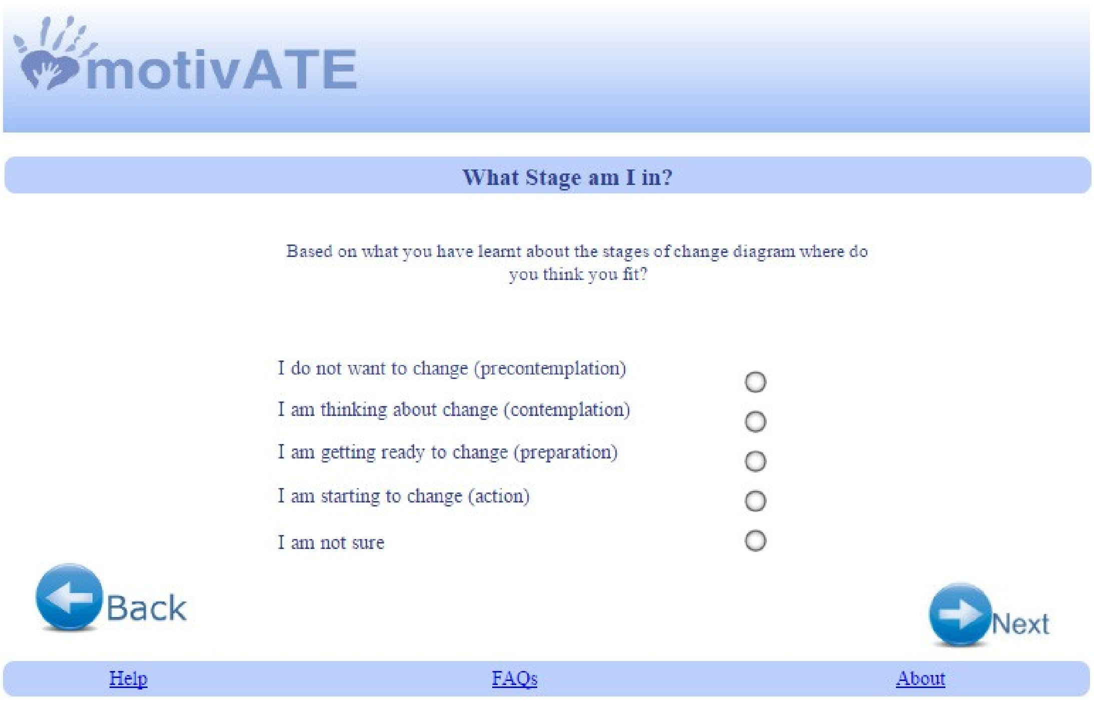

Module 2 Final Information

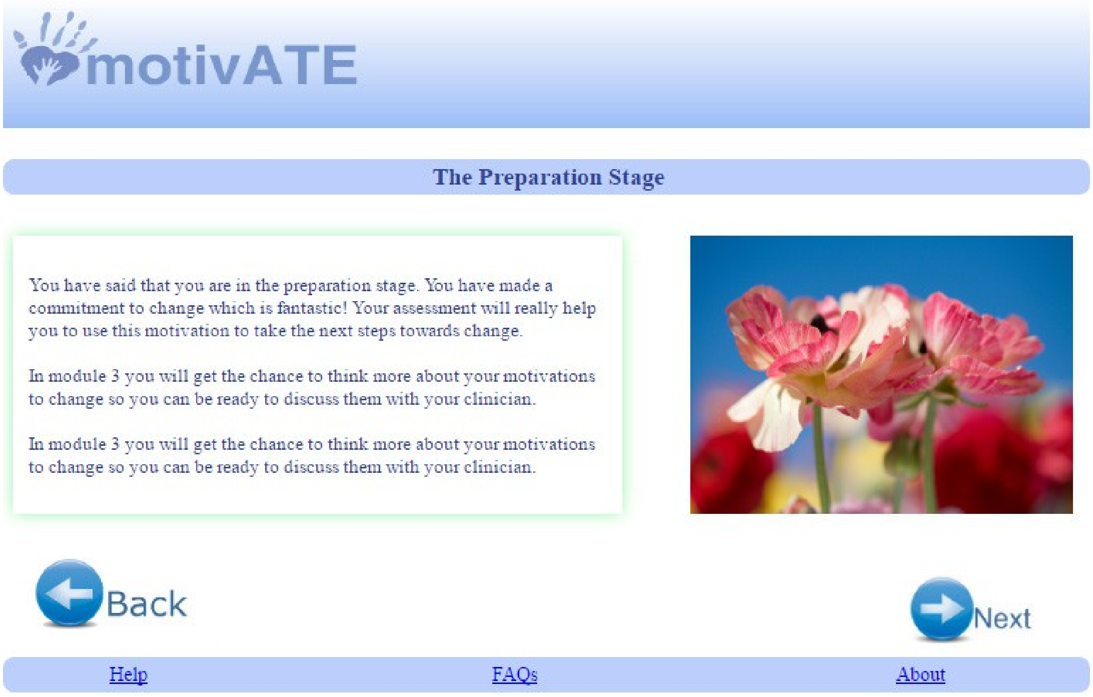

Module 3 Start

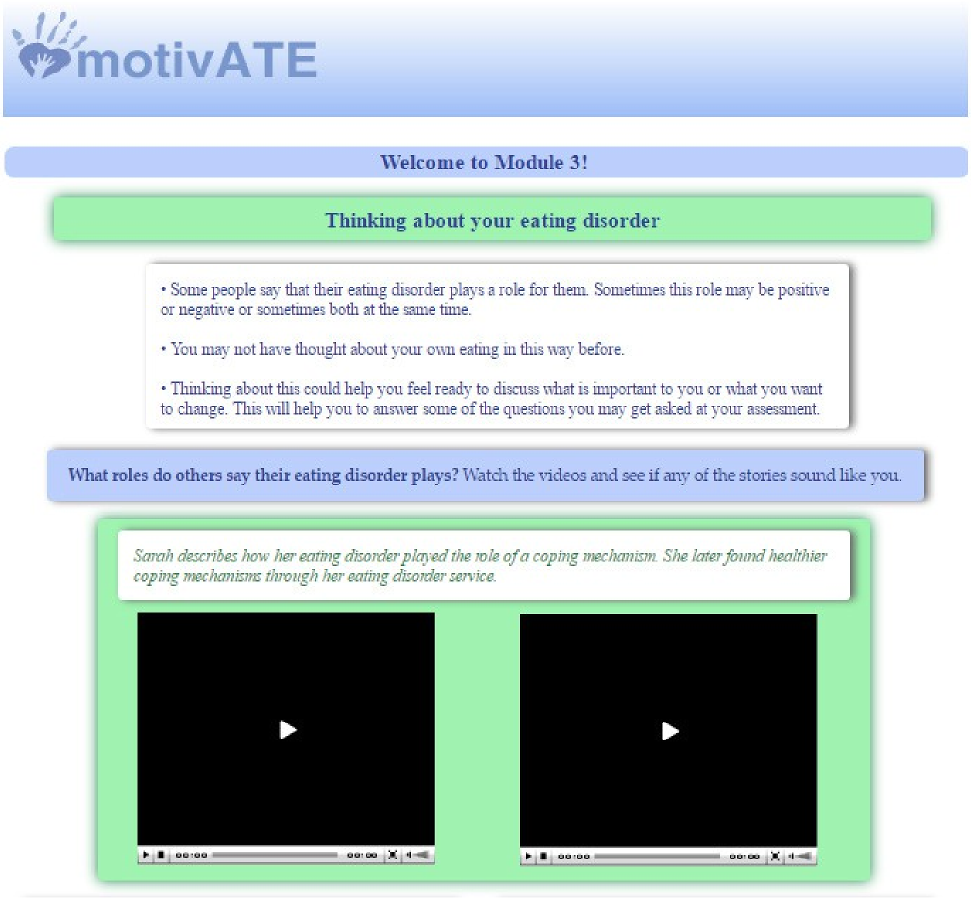

Module 3 Thinking About Your Eating Disorder

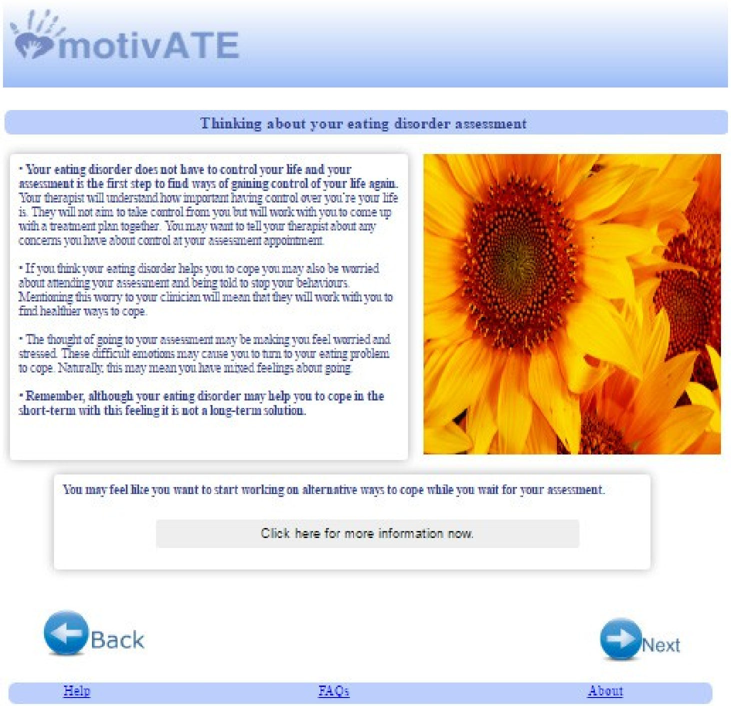

Module 3 Minnesota Study Information

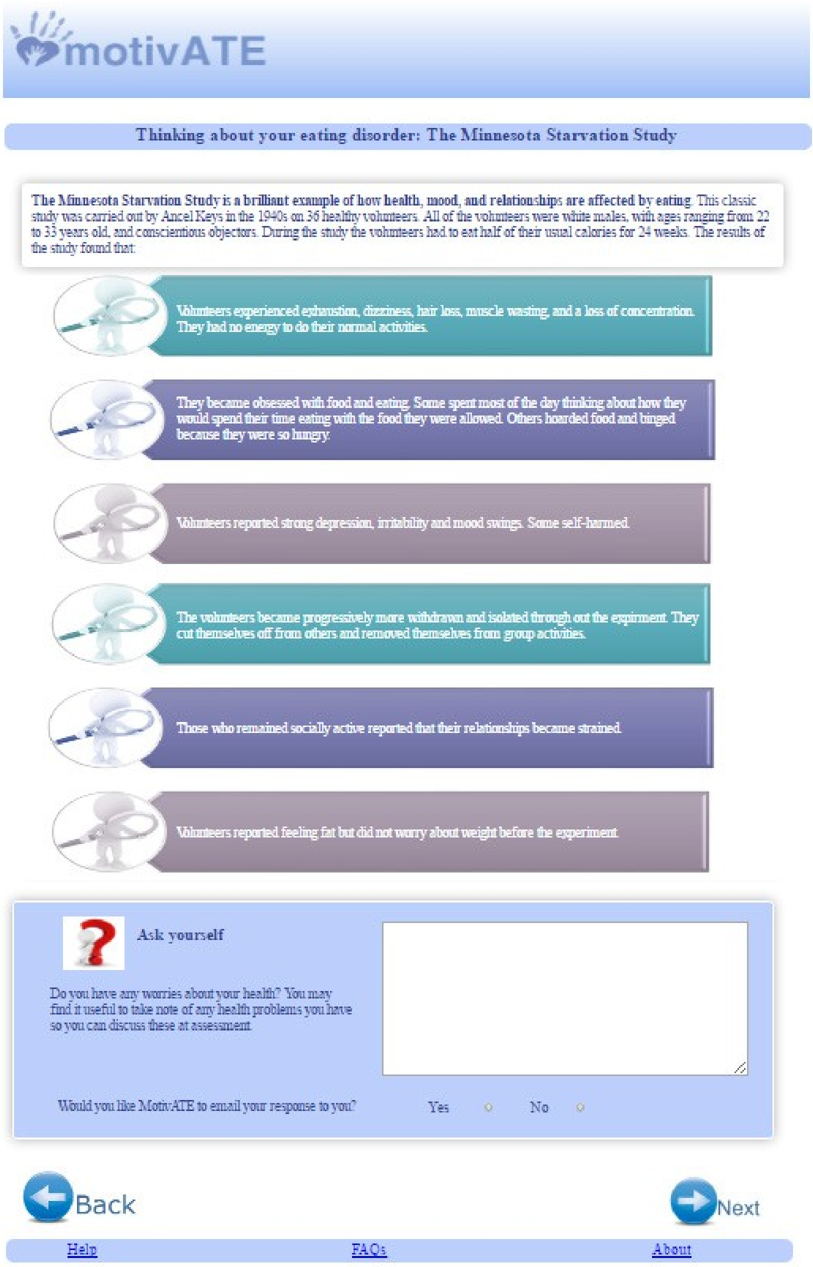

Module 3 Obsessional Thoughts Exercise

motivATE

Obsessional Thoughts and Eating

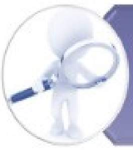

Studies show that people with eating disorders show more traits of an obsessive-compulsive personality than others. This means they may be more likely to:

- obsess about certain aspects of their life (e.g. food/weight)
- need strict rules and structure
- place higher importance on the need for control
- and have a higher need for perfection

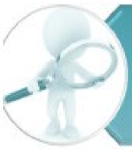

People with higher traits of perfection may see things in black and white which can be unhelpful. You may find yourself thinking in terms of success vs. failure without anything in between. This may spill into other aspects of your eating disorder (fat or thin, good or bad foods, eat nothing or eat everything).

These thinking styles can be unhelpful. Below are some examples of common all-or-nothing thoughts that may make you think twice about changing (an unhelpful thought).

Thinking through some of these unhelpful thoughts and replacing them with motivating thoughts may help you feel more confident on the day of your assessment.

Unhelpful thought 1: My therapist will think that I'm not ill enough.

MotivATing thought 1: You have been referred to an eating disorder service. This means that you, your family or your doctor think an assesament will be a good idea. Not feeling ill enough (or good enough) is an example of black and white thinking and may be related to low self-esteem which is common in people with an eating disorder. Your therapist can help you to address these thoughts.

Unhelpful thought 2: Going to the assessment and giving up my eating rules will be seen as a failure/ I'm worried about giving up my rules

MotivATing thought 2 : Nobody else would see this as a failure! Think of all the other achievements you could work towards if you weren't thinking about food all the time. Giving up the structure of rules may be difficult at first but with the support of your therapist you can see how freeing it is to focus on other aspects of your life!

Unhelpful thought 3: I need to be the thinnest that I can be. The eating disorder service will make me fat.

MotivATing thought 3: This is a classic example of black and white thinking. It is not realistic for 3 reasons.
1) the service will not make you do anything
2) there are many shapes and sizes in between fat and thin
3) it is impossible to be the thinnest person and also be healthy

Unhelpful thought 4: I'm never going to get better so what's the point in trying?

MotivATing thought 4: You may have previously tried to improve your eating behaviour by yourself and not been successful but that doesn't mean it's always going to be that way. There are so many different treatments out there for you to try and your therapist will help support you along the way and help work out a plan which will work best for you.

Ask yourself

- Do you find yourself thinking in all-or-nothing terms?
- Do you expect perfection?
- Do you worry too much about failing?
- Do you have lots of strict rules?

Try writing your own unhelpful and motivATing thoughts in the boxes to the right. These will be saved to your profile.

Unhelpful thought:

MotivATing thought:

Back Next

Help FAQs About

Module 3 Pros and Cons Exercise

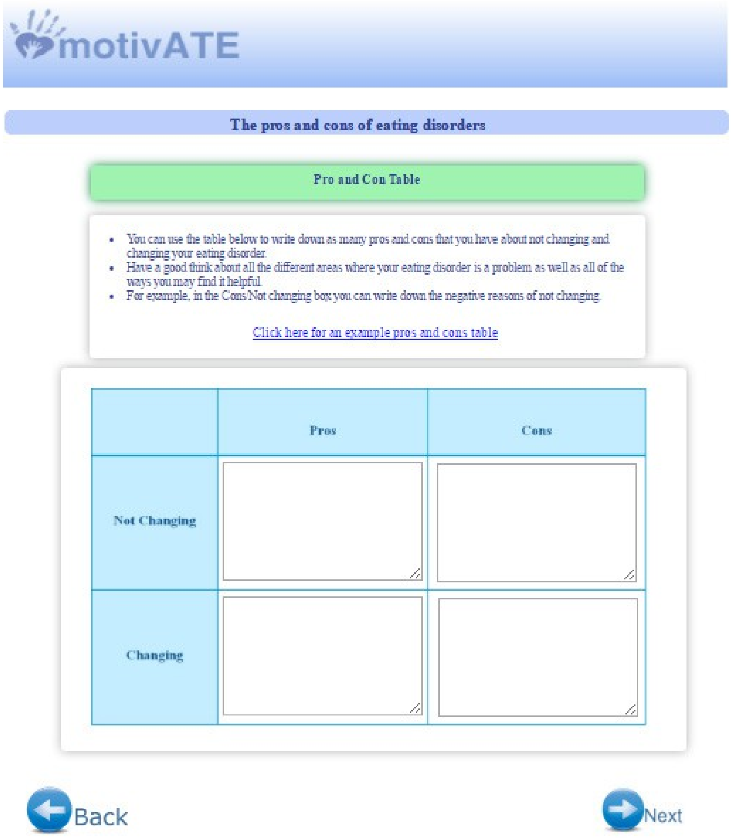

Module 4 Start

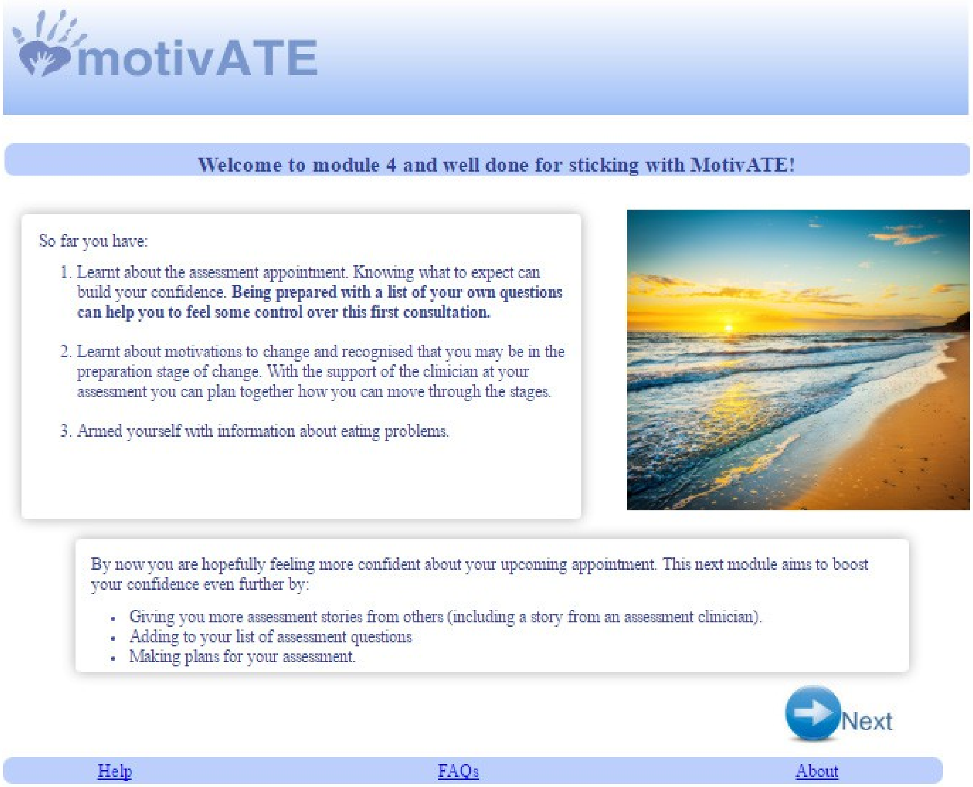

Module 4 Story Example

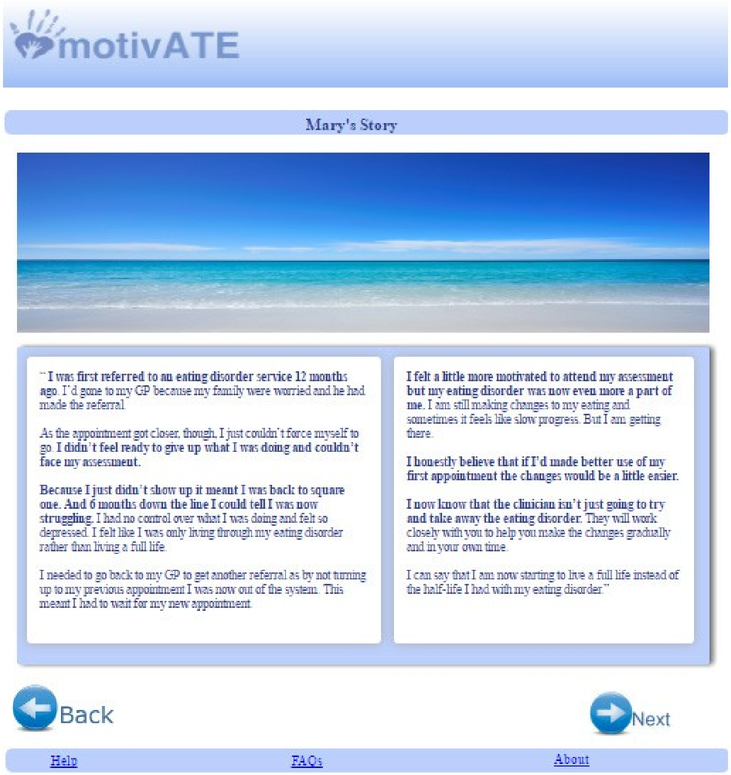

Module 4 Clinician's Perspective

motivATE

A clinician's thoughts on the assessment appointment

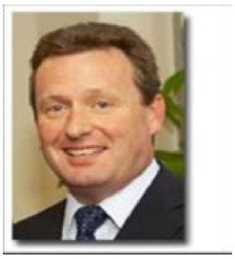

Hello my name is Ciarán and I work in an Eating Disorders Service offering assessment and treatment for people affected by eating disorders.

Most people are referred to our service by their GP, another professional and a few self refer.

I am aware that people can be nervous about their assessment and can have mixed feelings about being there as this is a common feature of those who come for assessment.

Therefore to help with knowing what is likely to happen at your assessment I have described below the major parts which you are likely to experience.

The clinician is trying to understand why you have come to see them. To help with this they will ask questions about your problem but this does not mean you have to answer every question and if you do not want to, then that is ok. **If you want to you can bring another person with you** and the clinician will only talk to them with your permission.

The assessment will cover:

- **Your eating behaviours and thoughts and how these affect you**, those closest to you, as well as all areas of your life such as work, education, hobbies and interests.
- **The history of the problem with food**
- **Any other areas that you think are relevant to how things are in your life** (for example low mood or anxious feelings).
- **The possible areas of risks to you.** These are areas where your health (physical and psychological) may be damaged by the impact of the behaviours/thoughts that you have. Assessing the risk is important to ensure your safety and that a full care plan can be agreed with you to reduce risk as much as possible.

You may be asked to complete questionnaires before the assessment. These may assess eating disorder behaviours and thoughts, or low mood but this depends on the service. If you have any questions about these you can ask the clinician in the assessment.

Indeed, at the assessment, I would encourage you to ask any questions you may have.
You may have questions about:

- Your eating disorder
- The service
- Likely treatments
- Any other areas you want to cover.

This will help build the best picture of what the problem is and guide towards what needs to be done to assist you in recovering. I would also encourage you to contact the Service before your assessment with questions or worries you may have.

I would also encourage you to use MotivATE to help prepare for the assessment and challenge any fears you may have about what might happen.

Overall the aim of the assessment is to understand what has brought you to the Service, what you want to do next and how the Service can help you to achieve what you want.

We know it takes courage to tackle eating disorders and with help you can overcome the impacts of your eating disorder and live a healthier more rewarding life.

Back

Next

Help FAQs About

Module 4 Assessment Questions Revisited

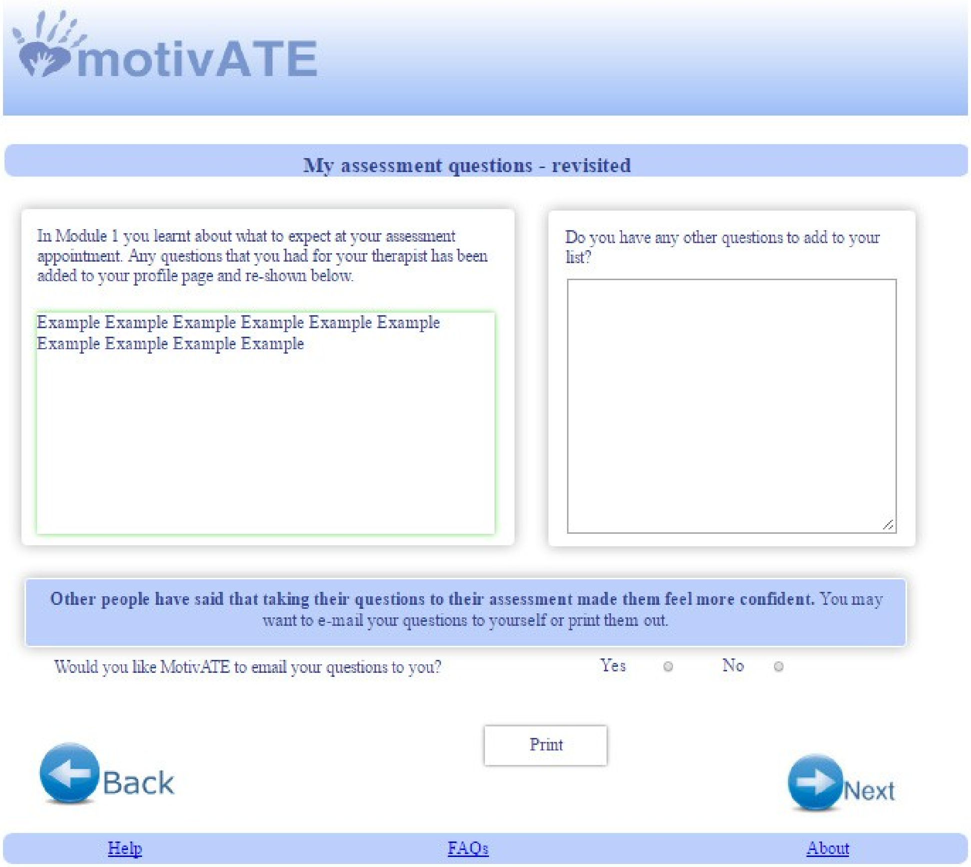

Module 4 Implementation Intentions Planning Exercise

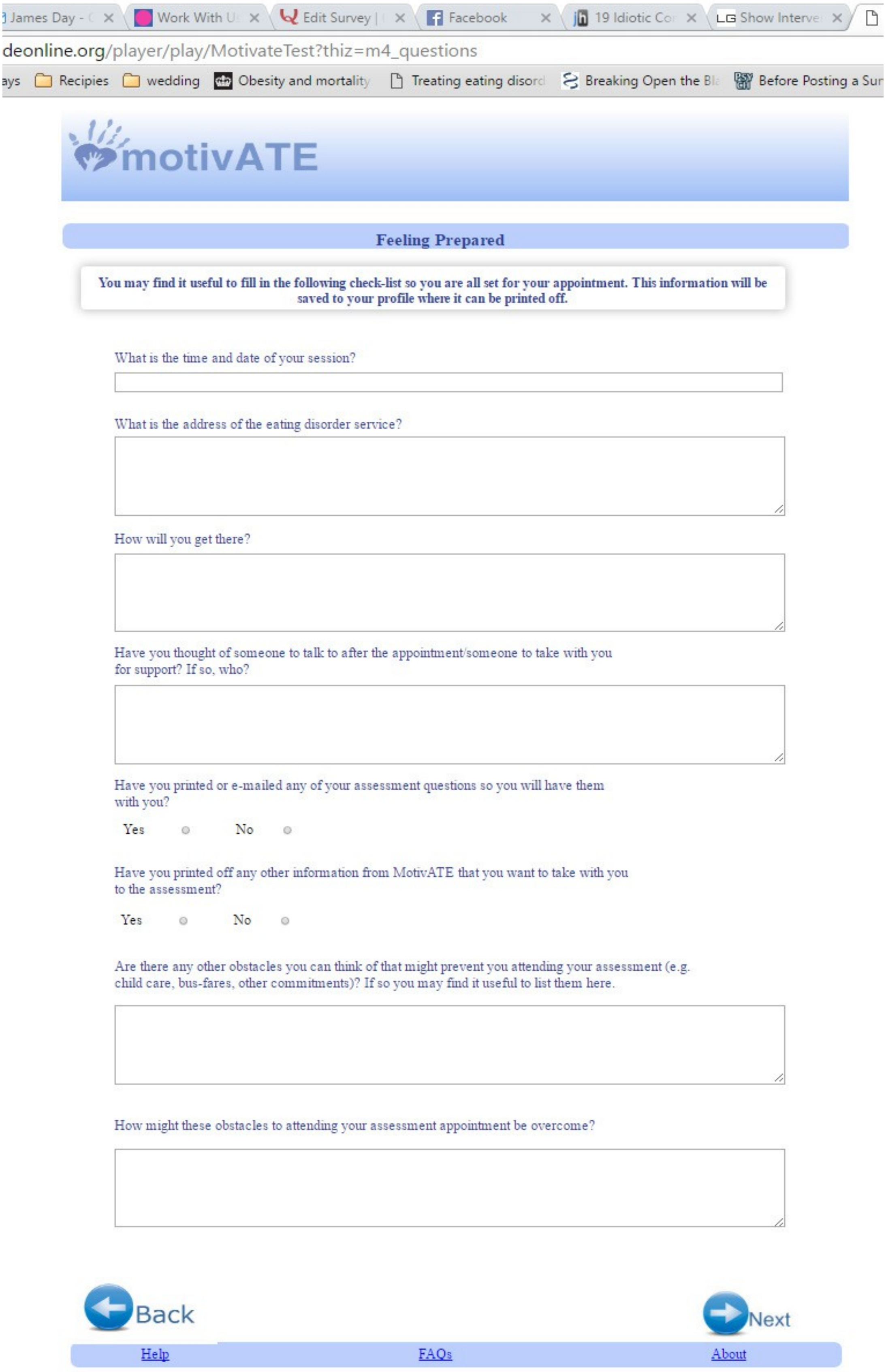

motivATE

Feeling Prepared

You may find it useful to fill in the following check-list so you are all set for your appointment. This information will be saved to your profile where it can be printed off.

What is the time and date of your session?

What is the address of the eating disorder service?

How will you get there?

Have you thought of someone to talk to after the appointment/someone to take with you for support? If so, who?

Have you printed or e-mailed any of your assessment questions so you will have them with you?

Yes No

Have you printed off any other information from MotivATE that you want to take with you to the assessment?

Yes No

Are there any other obstacles you can think of that might prevent you attending your assessment (e.g. child care, bus-fares, other commitments)? If so you may find it useful to list them here.

How might these obstacles to attending your assessment appointment be overcome?

Back Next

Help FAQs About

Profile Page

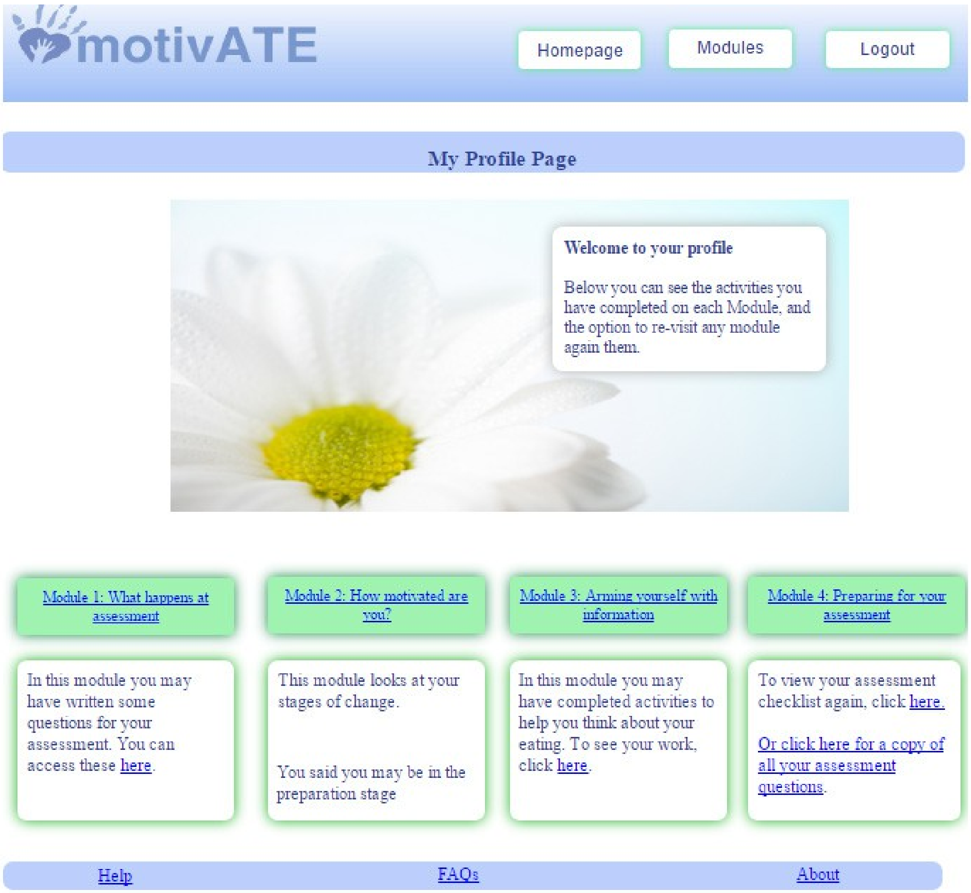

Example of Module 4 Profile Page

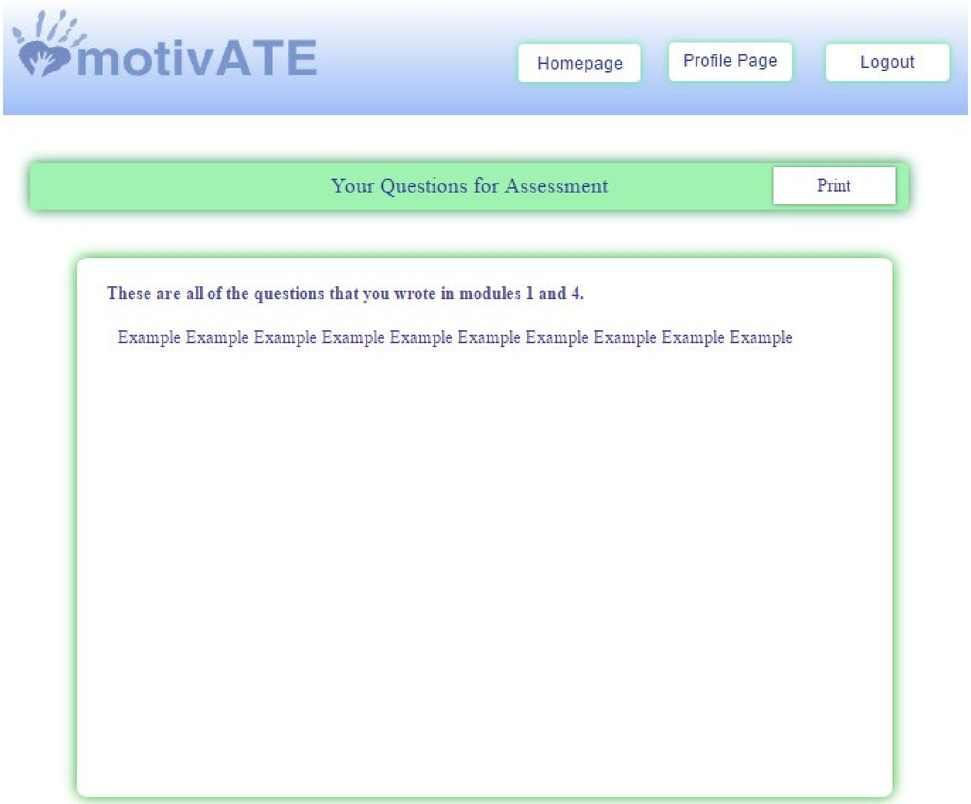

Supplement: Multimedia Appendix 1 [file jmir_v21i2e11874_app1.pdf]
