# Supplementary material for: A Web-Based Intervention (MotivATE) to Increase Attendance at an Eating Disorder Service Assessment Appointment: Zelen Randomized Controlled Trial
Source: J Med Internet Res. 2019 Feb 27;21(2):e11874. doi: 10.2196/11874 (PMC6414822; doi:10.2196/11874)
Supplement: Multimedia Appendix 2 [file jmir_v21i2e11874_app2.pdf]

Appendix 2: MotivATE Case Studies

The initial aim of this research was to recruit 20 participants who used MotivATE in order to conduct a full thematic analysis regarding the impact that MotivATE had on its users. However, potentially as a result of low uptake and engagement with the intervention as outlined above, only two people took part in the qualitative interviews. As such these are presented as case studies to provide some insight into user experiences of the MotivATE program. Both interview participants were female and took part in the interview after having both used MotivATE and attended their initial assessment appointments.

Case Study 1 - KP

In discussing her receipt of the invitation to use MotivATE, KP highlighted a potential contributing factor to the low uptake of the intervention, indicating that commonly she would discard additional pieces of paper that might come in the same letter. In this instance she did not, but it is possible that this view that accompanying material with the invitation letter was superfluous may have led participants to discard the letter inviting them to use MotivATE.

> *KP: "... there was two or three pieces of paper in there, and I normally discard them but I didn't, I took the time to read them on this occasion."*

Having read the invitation KP's initial feelings towards MotivATE seemed mixed. The novelty of the approach generated interest, however there were concerns about whether or not the tool would be useful and that they may simply constitute an 'NHS tick box' exercise.

> *KP: "I'd never had that with any other appointment so, umm, I was just interested to see if it was gonna be umm, tsch, something that was going to, that would encourage me, if it would give me any more information. Umm, where it was just a, kind of another NHS tick box, to be, you know full of legislation, or it was just a, whether it was actually going to be a helpful tool."*

However, despite these concerns KP indicated that MotivATE proved to a useful tool, particularly in the immediate run up to the assessment appointed. KP mentioned that she used MotivATE twice, firstly going through it quite quickly in such a way that she did not take much in, but then returning to it in the days before her appointment as she found that MotivATE helped to keep her calm and in control.

> *KP: "... it helped me feel a bit calmer before I got there and stuff."*

When asked whether she felt MotivATE contributed to her decision to attend the assessment, KP highlighted this as a potential contributor to her attendance.

> *KP: " yeah I think it probably, because I was quite, really quite anxious the day before, umm, by, um, by kind of revisiting it ... it stopped me getting out of control in my mind and making it into something much bigger than it actually was."*

KP attributed this impact on her primarily to the more practical elements of MotivATE, including encouraging the user to think about their questions and make notes (though KP indicated she did not make actual notes, she suggested simply thinking through her answers was useful) and addressing possible preconceptions regarding the assessment. This she said helped reduce anxieties by focusing

on the reality of the situation rather than what she called 'the fluffy stuff'; which she defined as the unnecessary concerns she had before the assessment, such as worries over *'the use of phrases and terminology that I'm not going to understand'* and the idea that '*it's going to be some big, dramatic, me sat in front of a panel of three doctors asking me questions*'.

> *KP: "I think it lessened my anxieties because it, umm, it kind of, made it seem more practical and useful and kind of maybe tool based, rather than talking about all the fluffy stuff"*

Though her overall experience of MotivATE was positive, KP did have a number of suggestions for improvements. These primarily related to the design, particularly of the front page, which she felt was somewhat confusing in places in regards to the intent and focus of MotivATE, as well as the design and placement of the logos. She also indicated intervention could be updated to make it look less old fashioned, though she re-iterated that she felt the content of MotivATE was good.

> *KP: "... it looks a bit old fashioned. The kind of, the font and the way it's written. But the actual content is good."*

Overall the experience of KP presents a positive picture of the impact of MotivATE when delivered prior to the assessment appointment, suggesting that in at least one case it successfully reduced participants' anxieties about the assessment appointment and contributed to her decision to attend. However as noted above, improvements can be made to the design, as well as the way in which it is offered, which could indicate ways in which uptake could be improved in future iterations.

<u>Case Study 2 - ET</u>

As with KP, ET noted the arrival of the MotivATE intervention letter along with the assessment letter, suggesting that this created a clear association between the two. In this case it appears that ET was already intending to go to her assessment appointment and that this intent carried over to the use of MotivATE.

> *ET: "The invitation for my appointment at my invitation to use MotivATE came at the same time, erm, so I felt it kind of went hand in hand and if I was going to go to the interview I may as well use the MotivATE program"*

Despite her intent to use the intervention, KP noted that the invitation to use MotivATE raised a number of concerns. Primarily she felt that the offer to use MotivATE indicated that she had been placed in a preconceived category rather than being treated as an individual, and that the intervention would simply be a 'one size fits all' solution. ET had the similar concern to KP that this simply represented a 'tick box' exercise rather than a genuine attempt to offer a useful and effective pre-treatment tool.

> *ET: "One of my worries was that I was going to be put into a category and it kind of felt like I was already being categorised. I was already kind of in a, one size fits all tick box kind of situation."*

When discussing using MotivATE ET presented a much more negative view of her experience than KP, suggesting that the approach taken by MotivATE felt patronizing. ET felt that the intervention should

have been much more direct, and that the current approach was too gentle and took too long, resulting in the feeling that it was wasting her time.

> *ET: "I noticed it was kind of like easing me into it, and then it got to the specific stuff. And I felt kind of like, a bit patronized by that because I was like if you're going to ask me these questions, which I know you're in inevitably going to do because that's what the software is for like, I that's what questionnaire thing is for. You might as well just ask me straight away rather than just have to make me go through all these questions"*

> *ET "it was just it was kind of wasting my time ... it is just too long."*

Perhaps even more concerning was that ET reported that the exercises in MotivATE actually increased her sense of anxiety regarding the assessment, both by reinforcing her initial concerns of being arbitrarily placed in a category and as a result of concerns that information she gave to MotivATE might be used to catch her out in the assessment should what she say not completely match what she wrote down. As a result of this when asked what impact MotivATE may have had on her decision to attend ET responded that it had actually had a negative impact.

> *ET: "I was putting in answers to things umm, that I assumed the person who was assessing me was going to read, I felt like I had to be careful what I would then say because I thought, what if I accidentally say something that doesn't completely marry up to what I said in my MotivATE thing"*

Despite this ET did note towards the end of the interview that she believed the concept behind MotivATE was a good idea, but that it needed more work and adjustment before she felt it was something that would be a benefit to users such as herself prior to the assessment appointment.

> *ET: "I think MotivATE is a good idea. And I think it's got a good concept behind it, it just needs adjusting a little bit..."*

This gives a very different perspective to the experiences of KP, and highlights how careful future iterations of MotivATE must be to not only be supportive of individuals in earlier stages of change, who are uncertain about attending their assessment, but to also ensure that it does not put off those who are more intent and ready to take that step.
